# Lymphomatoid Granulomatosis in HIV-2: A Rare Entity

**DOI:** 10.7759/cureus.19992

**Published:** 2021-11-29

**Authors:** Clara Matos, Ana Gonçalves, Susana G Pereira, Sofia Carola, Teresa Branco

**Affiliations:** 1 Internal Medicine, Hospital Prof. Doutor Fernando Fonseca, Amadora, PRT; 2 Clinical Hematology, Hospital de Santo António dos Capuchos, Centro Hospitalar Universitário de Lisboa Central, Lisbon, PRT; 3 Oncology, Instituto Português de Oncologia de Lisboa Francisco Gentil, Lisbon, PRT

**Keywords:** chemotherapy, human immunodeficiency virus type 2, epstein-bar virus, lymphoproliferative disorder, lymphomatoid granulomatosis

## Abstract

Lymphomatoid granulomatosis (LYG) is a rare B-cell lymphoproliferative disorder associated with Epstein-Barr virus (EBV) infection and is frequently associated with immunodeficiency. Pulmonary involvement with angiocentric distribution is the most common clinical manifestation. Diagnosis is confirmed by tissue biopsy, usually from lung lesions. Due to the paucity of reported cases, there is no validated treatment for LYG. Therapeutic options include interferon-alpha, systemic corticosteroids, rituximab, chemotherapy, and autologous hematopoietic stem cell transplantation. We report a case of a 49-year-old man, with human immunodeficiency virus type 2 (HIV-2) infection, who was diagnosed with LYG with lung involvement and had a full remission after treatment with R-CHOP (rituximab, cyclophosphamide, doxorubicin, vincristine, and prednisone).

## Introduction

Lymphomatoid granulomatosis (LYG) is a rare B-cell lymphoproliferative disorder associated with Epstein-Barr virus (EBV). Prevalence is unknown as there are only 600 cases reported in the literature. LYG has a predominant extra-nodal involvement, affecting primarily the lungs (80%) [1], followed by skin, kidney, liver, and central nervous system (CNS) [1,2]. Lymph node and bone marrow involvement are extremely rare [3]. Pulmonary involvement is characterized by multifocal nodules and nodular masses with an angiocentric distribution [1,3]. Patients are usually symptomatic, presenting with respiratory and constitutional symptoms, such as cough, dyspnea, chest pain, fever, weight loss, and fatigue.

There is a universal association with EBV, which likely plays an important role in the pathophysiology of LYG [4]. Serologic tests generally show evidence of prior EBV infection [3]. LYG is frequently associated with underlying immunodeficiencies, such as HIV infection, autoimmune disease, organ transplantation, Wiskott-Aldrich syndrome, amongst others [1,2].

Differential diagnosis includes Wegener granulomatosis, Churg-Strauss syndrome, tuberculosis, sarcoidosis, histoplasmosis, coccidioidomycosis, and lymphoma, amongst other multisystemic disorders with lung involvement [5].

LYG diagnosis is confirmed by tissue biopsy, usually from lung lesions, where angiocentric and angiodestructive polymorphous lymphoid infiltrates can be seen, consisting of EBV-positive B cells with an inflammatory background. Staging is defined by the amount of EBV-positive B cells per high-power field (HPF): grade 1 has <5 EBV-positive B cells/HPF; grade 2 has 5-20 EBV-positive B cells/HPF; grade 3 has >20 EBV-positive B cells/HPF and may be associated with extensive necrosis [1,2].

As a rare disease, there isn’t a gold-standard treatment for LYG. In patients with low-grade disease and reversible immunosuppression, an expectant course of action can be adopted, and, in some patients, spontaneous regression is observed. Therapeutic options range from interferon-alpha, systemic corticosteroids, rituximab, chemotherapy, and autologous hematopoietic stem cell transplantation [3].

LYG is generally associated with a poor prognosis. It can lead to respiratory failure, which is the most frequent cause of death, neurological complications, infection, or progression to aggressive B-cell lymphoma [1].

## Case presentation

We present the case of a 49-year-old man, born in Cape Verde, residing in Portugal for the past 18 years with type 2 diabetes and human immunodeficiency virus type 2 (HIV-2) infection, diagnosed in 2010, at A2 stage according to the CDC Atlanta classification (nadir of 310 CD4+ cells/mL in 2015). Antiretroviral therapy (ART) was not started at the time of diagnosis due to alcohol abuse and a lack of adherence to therapy and medical care.

**Figure 1:**
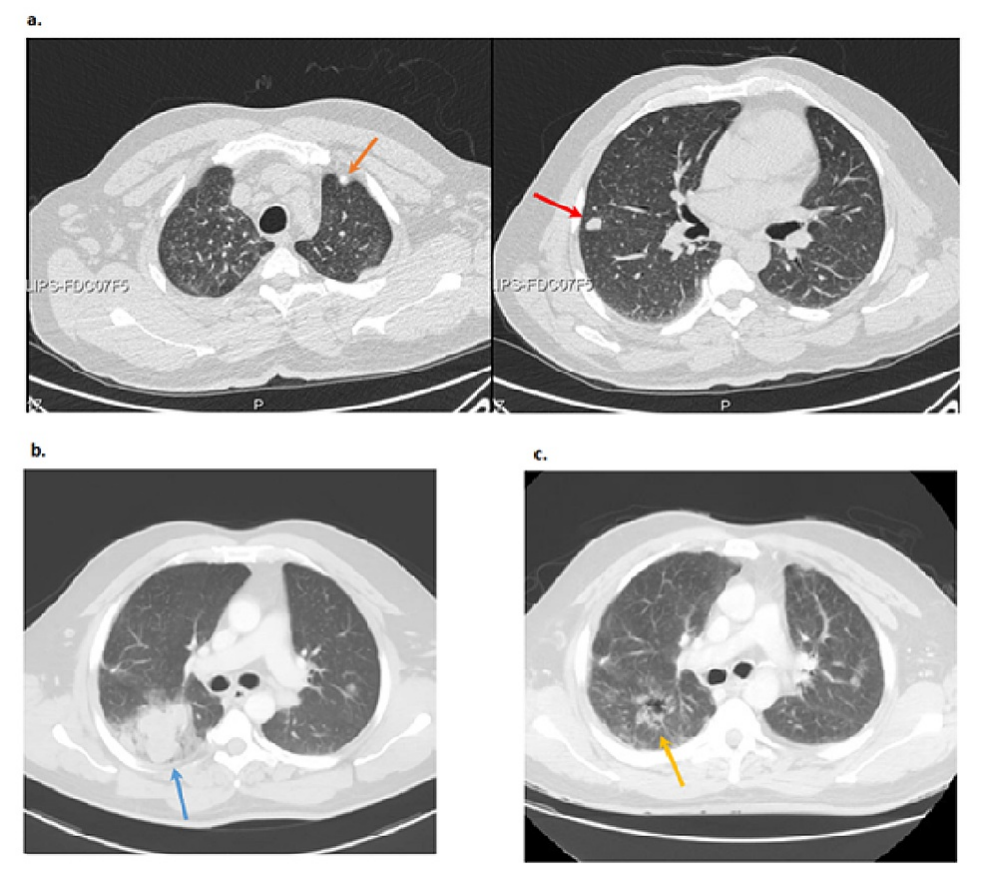
Evolution of LYG with lung involvement, before (a, b) and after treatment (c) a. Initial chest CT scan shows nodules in the anterior segment of the upper left lobe with 9mm diameter (orange arrow) and in the medium right with 12mm diameter (red arrow) b. Chest CT scan at six-month follow-up shows a localized mass in the apical segment of the inferior right lobe, with 55 x 39 mm (blue arrow) c. Chest CT scan at 18-month follow-up (six months after R-CHOP) shows regression of the mass previously seen in the inferior right lobe (yellow arrow) LYG: lymphomatoid granulomatosis; R-CHOP: rituximab, cyclophosphamide, doxorubicin, vincristine, and prednisone

The evolution of LYG with lung involvement, before and after treatment, is depicted in Figures 1a-1c. In March 2017, the patient was admitted to the emergency department due to pneumonia with hypoxemia. Chest computed tomography (CT) scan revealed multiple axillary and mediastinal enlarged lymph nodes, a diffuse micronodular infiltrate, and large nodules in the anterior segment of the left upper lobe and in the middle lobe (Figure 1a).

Bronchoscopy was performed, which proved to be inconclusive, requiring a wedge resection of the middle lobe. The anatomopathological results revealed grade 2 LYG. Serologic testing showed positive Epstein Barr nuclear antigen (EBNA) and viral capsid antigen (VCA) immunoglobulin G (IgG) and negative VCA IgM, translating previous exposure to EBV. HIV-2 viral load was up to 1948 copies/mL and CD4+ count was 428 cells/mL. The patient was started on ART with emtricitabine/tenofovir disoproxil fumarate 200mg/300mg q24h and lopinavir/ritonavir 400mg/100mg q12h, and was referred to HIV and hematology outpatient consultations. He maintained good adherence to the medication and in three months, he had a suppressed HIV-2 viral load (<40 copies/mL) and higher CD4+ count of 650 cells/mL.

In November 2017, the patient complained of worsening fatigue and dyspnea. Chest CT scan revealed a large mass located predominantly in the apical segment of the right lower lobe with necrotic areas, and multiple pulmonary nodules bilaterally distributed (Figure 1b). CT-guided transthoracic biopsy was performed, and pathology revealed grade 3 LYG. Immunohistochemical study revealed expression of CD20, B-cell lymphoma 2 (BCL2), BCL6, and NUM1. Considering disease progression, despite improved immunological status on ART, the patient was started on combination chemotherapy with R-CHOP (rituximab, cyclophosphamide, doxorubicin, vincristine, and prednisone), completing eight cycles. Follow-up chest CT scan performed in September 2018 showed a total regression of the mass and dimensional reduction of the bilateral pulmonary nodules (Figure 1c). Due to structural lung sequelae, he was referred to pulmonology consultation and started domiciliary oxygen therapy. A positron emission tomography (PET) scan performed in June 2020 showed no recurrence of the disease. In a follow-up evaluation in July 2021, the patient remained asymptomatic.

## Discussion

LYG is a rare disorder, which is usually associated with immunosuppression. HIV-2 infection has a more indolent course when compared to HIV-1, however, if left untreated, it will present with the same complications. HIV-2 infection is particularly relevant in Portugal, due to its historical and still-standing socioeconomic relationship with West African countries. There are 2030 HIV-2 infections reported in Portugal, representing 3.3% of all HIV infections [6].

Dysregulation of T cell function in HIV infection, even though predominantly involving CD4+ cells, can give leeway to unchecked EBV activity and proliferation and contribute to the pathophysiology of LYG [3]. To the best of our knowledge, there has only been one previously reported case of LYG associated with HIV-2 infection, where the patient presented with CNS involvement and ART was started with stabilization of the lesion [7].

In the case of our patient, considering the diagnosis of a grade 2 LYG and a rather low disease burden, treatment of HIV-2 infection and active surveillance was considered a valid course of action. However, disease progression was observed despite improved immunological status. Combination chemotherapy with rituximab is one of the treatment options for high-grade LYG, similar to B-cell lymphoma. In a series of 11 patients with LYG, five were treated with R-CHOP resulting in an average progression-free survival of 22.4 months and overall survival of 50 months [8]. There have been other reports of the use of R-CHOP in the treatment of high-grade LYG, with variable response [9-13]. Our patient was treated with eight cycles of R-CHOP with an excellent response, maintaining a complete remission at three years of follow-up.

## Conclusions

LYG is a rare and potentially fatal disease, for which effective treatment options are still scarce. This case highlights the importance of a thorough differential diagnosis of lung lesions, particularly in an immunocompromised host. It could also showcase the importance of a paradigm change in the treatment of HIV infection, with emphasis on the early initiation of ART in all patients, independent of staging. Lastly, this case stands out for the favorable response to R-CHOP, which may strengthen its role in the treatment of high-grade LYG.
